# Resilience And Coping: The Chicken and The Egg Paradox

**DOI:** 10.1192/j.eurpsy.2022.510

**Published:** 2022-09-01

**Authors:** N. El Ouni, A. Braham, O. Charaa, H. Kalboussi, J. Maatoug, N. Mrizak

**Affiliations:** 1 Farhat Hached University Hospital, Psychiatric Research Laboratory Lr12es04, sousse, Tunisia; 2 Farhat Hached University Hospital, Occupational Health Department, sousse, Tunisia; 3 Farhat Hached University Hospital, Department Of Epidemiology, sousse, Tunisia

**Keywords:** Stress, resilience, coping, Coronavirus

## Abstract

**Introduction:**

Since the outbreak of the 2019 coronavirus, healthcare workers found themselves on the front lines of an unprecedented battle. Being characterized by adversity, this experience represents a fertile ground for the study of resilience.

**Objectives:**

Our study aims to clarify the phenomenon of resilience through its influence on perceived stress level and its connection with coping strategies.

**Methods:**

A cross-sectional study was conducted involving 254 healthcare professionals in the region of Sousse during the pandemic. In addition to socio-demographic and professional characteristics, Resilience, perceived stress, and coping strategies were assessed using the Connor-Davidson Resilience Scale (CD-RISC), the PSS10 scale, and the Brief Cope questionnaire, respectively.

**Results:**

The overall mean [±standard deviation (SD)] age of the participants was 32.9 ± 8.76 years with a sex ratio (M / F) of 0.51. The assessment of resilience among participants revealed a mean score of 64.99 ± 14.72. The majority of participants evinced a score> 50 (82.68%) and 39.76% had a score >70. Our results revealed that, on the one hand, problem-focused coping strategies were positive predictors of resilience (p<10^-3^), accounting for 3.6% of its variance. On the other hand, coping strategies (problem-focused strategies and avoidance strategies) are also an integral part of the process by which resilience significantly influences the level of perceived stress (mediating factor).

**Conclusions:**

Despite its complexity, the relationship between resilience and coping strategies is undeniable and it is a part of an important line of intervention opening the way to better identifications and care.

**Disclosure:**

No significant relationships.

